# The role of sex in multiarterial coronary artery bypass grafting: A landmark and risk factor analysis

**DOI:** 10.1016/j.xjon.2026.101847

**Published:** 2026-04-30

**Authors:** Joseph Kletzer, Albi Fagu, Laurin Micek, Matthias Eschenhagen, Maximilian Kreibich, Martin Czerny, Kilian Franke, Tau Hartikainen, Mirjam G. Wild, Ulrich Franke, Tim Berger

**Affiliations:** a Department of Cardiovascular Surgery, University Heart Center Freiburg - Bad Krozingen, University Medical Center Freiburg, Freiburg, Germany; b Faculty of Medicine, University of Freiburg, Freiburg, Germany; c Department of Cardiology and Angiology, University Heart Center Freiburg - Bad Krozingen, Bad Krozingen, Germany

**Keywords:** sex differences, CABG, internal thoracic artery, landmark comparison, risk differences

## Abstract

**Objective:**

Bilateral internal thoracic artery (BITA) grafting is associated with superior long-term outcomes in coronary artery bypass grafting (CABG), yet sex-based disparities persist, with women facing greater early morbidity and mortality.

**Methods:**

This retrospective, single-center cohort study included 2010 patients undergoing CABG with BITA grafts between 2005 and 2024, stratified by biological sex. The primary outcome was a composite of death and repeat coronary revascularization. Analyses included weighted survival curves, landmark analyses excluding early events, competing risk regression by sex, and random survival forests to characterize sex-specific risk profiles.

**Results:**

Of 3872 patients undergoing total CABG and 2010 patients undergoing BITA-CABG, 268 (13.3%) were female. Women were older (68.0 vs 65.0 years, *P* < .001), had smaller graft diameters (left internal thoracic artery and right internal thoracic artery both *P* < .001), greater perioperative risk (European System for Cardiac Operative Risk Evaluation 1.40 vs 0.99, *P* < .001), and smoked less (26% vs 44%, *P* < .001). Postoperative mortality (23% vs 15%, *P* = .003) and reintervention (15% vs 11%, *P* = .049) rates were greater for women. After adjustment, the composite outcome disparity was minimized (log-rank *P* = .051); long-term survival and revascularization rates were similar in the 30-day landmark-analysis (*P* = .139). In women, outcomes were most strongly influenced by New York Heart Association class, pulmonary hypertension, and graft flow; whereas in men, the main predictors were smoking, previous infarction, and peripheral artery disease.

**Conclusions:**

Early outcome disparities after BITA-CABG are driven by anatomical and clinical factors in women, including smaller conduit and vessel size and greater perioperative risk, particularly in the immediate postoperative period. Long-term outcomes are similar between sexes once this phase is overcome. Distinct risk profiles underscore the need for individualized, sex-specific management in contemporary CABG care.

**Figure:**
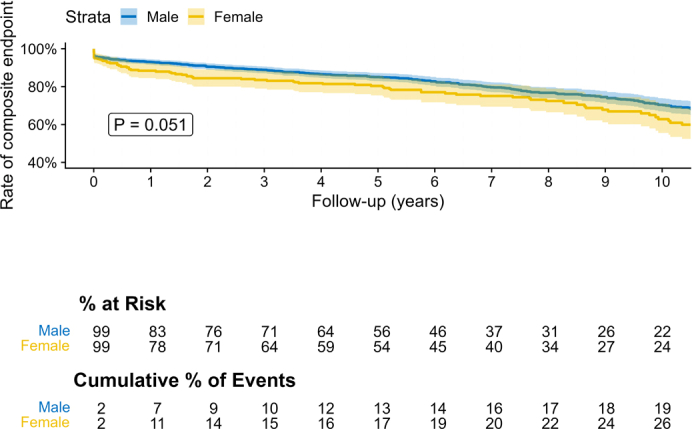
Composite outcome of death and coronary revascularization during follow-up.

**Central Message**

Although outcome disparities seem to vanish after adjusting for baseline differences, the sexes seem to be affected differently by the same risk factors, making individualized care even more critical.

**Perspective**

This study suggests that, when patients are carefully selected for bilateral internal thoracic artery grafting, sex-based differences in CABG outcomes are markedly reduced, supporting more equitable use of multiple arterial grafts in women.

Coronary artery bypass grafting (CABG) remains a cornerstone of revascularization for patients with advanced coronary artery disease, providing robust long-term survival benefits across diverse populations.^1^^,^^2^ In observational studies, bilateral internal thoracic artery (BITA) grafting has been associated with lower rates of repeat revascularization and possibly improved long-term outcomes compared with single internal mammary artery or venous conduits, with analysis supporting its expanding role in surgical practice. However, these data are limited by bias, and the only large randomized trial did not demonstrate a clear survival advantage in the intention-to-treat analysis.^3^, ^4^, ^5^ In addition, persistent sex-based disparities in CABG outcomes continue to raise clinical concerns: women consistently experience greater rates of postoperative morbidity and mortality than men.^6^, ^7^, ^8^ The underlying causes of these sex-based disparities are complex and multifactorial. Women undergoing CABG tend to be older, have smaller vessel diameters, and present with greater rates of anemia and frailty, all of which might add technical complexity and susceptibility to early postoperative complications.^9^, ^10^, ^11^

Against this background, the present study aims to systematically evaluate sex differences in long-term outcomes and risk factor profiles among patients undergoing BITA-CABG, providing new insights to guide sex-specific prognostication and personalized care. Specifically, we aimed to determine whether sex-based disparities persist in the long term after accounting for baseline risk and early postoperative events and to delineate sex-specific risk profiles among patients undergoing BITA-CABG.

## Methods

### Ethical Statement

Institutional review board approval for this study (25-1208-S1-retro) was granted by the Ethics Committee of the University of Freiburg on August 19, 2025. Because of the retrospective nature of the research, the requirement for obtaining individual informed consent from participants was waived.

### Data Collection

In this retrospective, single-center study, all patients who underwent isolated CABG with BITA grafts at the University Heart Center Freiburg - Bad Krozingen between January 2005 and December 2024 at the center were included. Clinical data were comprehensively extracted from electronic patient records, and specialized diagnostic data, such as coronary angiograms, were reviewed by experienced clinicians. Of the initial 3872 consecutive patients who underwent CABG, 2010 remained after we excluded those not treated with BITA grafting. These patients formed the final study cohort and were subsequently stratified by their biological sex for further analyses. As a grafting strategy at our center, the left internal thoracic artery (LITA) is routinely grafted to the left anterior descending artery, whereas the right internal thoracic artery (RITA) is most commonly used as a free or Y-graft to supply additional lateral or inferior coronary targets. Due to restrictions by our local ethics committee, raw data used for in this manuscript cannot be shared.

### Definition of Primary Outcome Measure and Model Parameters

The primary outcome was a composite of all-cause death during follow-up and repeat coronary revascularization, including percutaneous coronary intervention and surgical intervention. Mortality data were verified using the official government registry, ensuring accuracy, and revascularization events were identified from electronic patient records, providing precise follow-up information. Graft size was measured using duplex sonography. Intraoperative graft flow was measured using transient time flow measurement probes (QuickFit TTFM Probe; Medistim). Graft size was measured preoperatively using duplex sonography.

### Statistical Analysis

Statistical analyses were conducted using R (version 4.3.3) with RStudio. For data visualization, we used ggplot2 (version 3.5.0). Normality was assessed via Shapiro-Wilk test; normally distributed variables were summarized as means ± standard deviation and compared using *t* tests, whereas non-normal variables were reported as medians with interquartile range and compared using Mann-Whitney *U* tests. Categorical data were analyzed with χ^2^ or Fisher exact tests.

**Figure E1:**
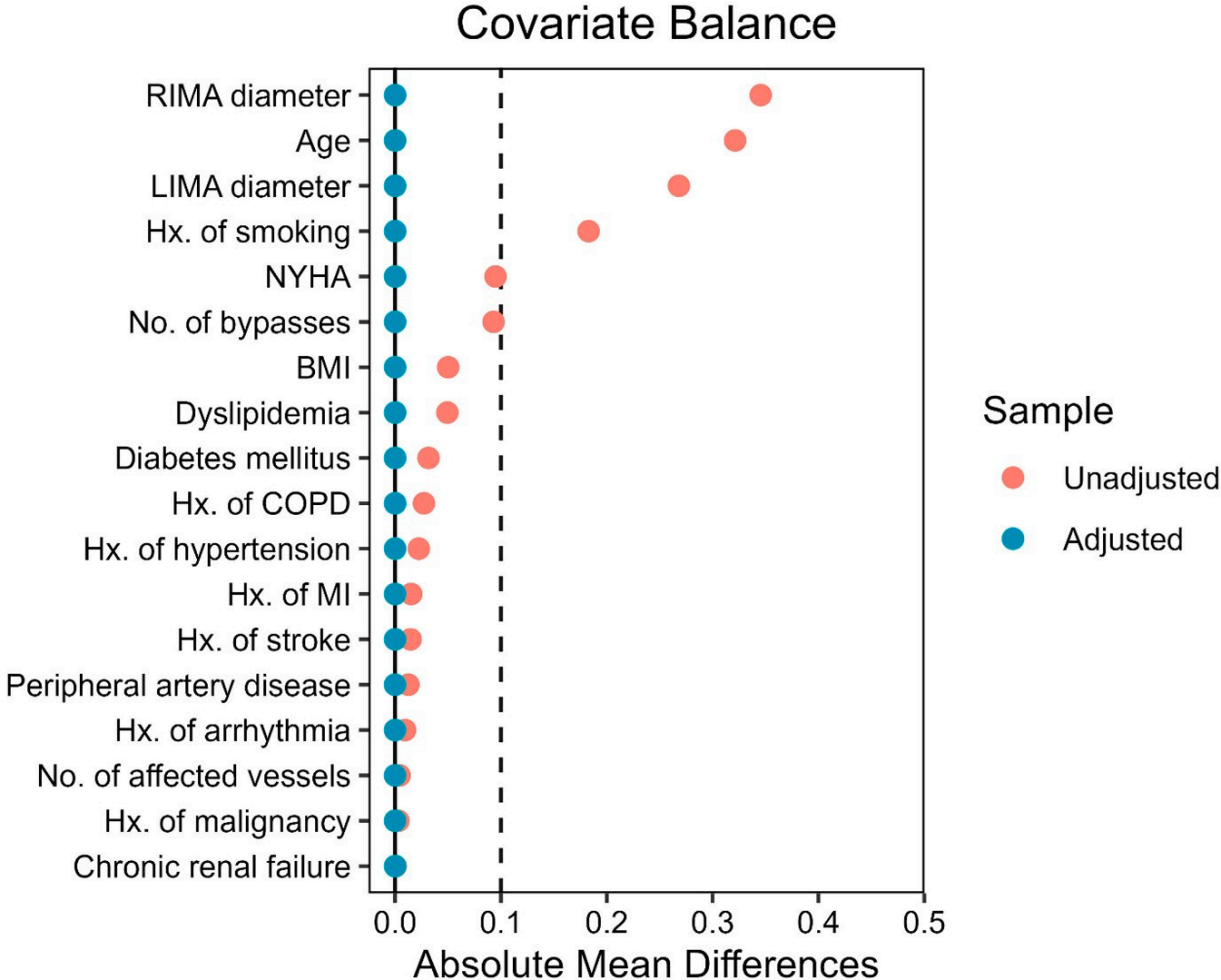
Love plot showing the absolute mean differences of the study cohort before and after weighing using the overlap weighing method. *RIMA*, Right internal mammary artery/same as right internal thoracic artery; *LIMA*, left internal mammary artery/same as left internal thoracic artery; *Hx*., history; *NYHA*, New York Heart Association; *BMI*, body mass index; *COPD*, chronic obstructive pulmonary disease; *MI*, myocardial infarction.

**Figure E2:**
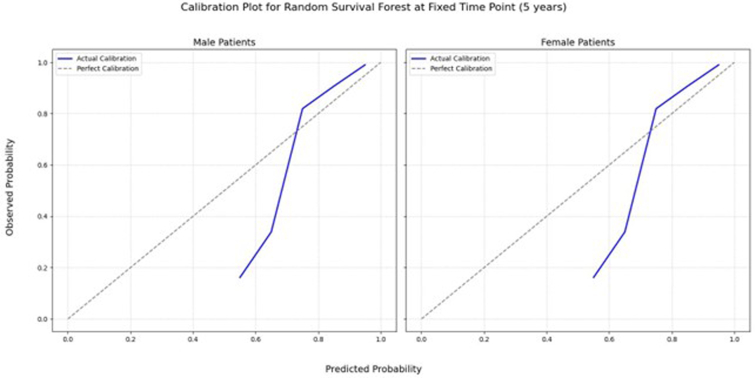
Calibration plot for composite outcome.

**Figure E3:**
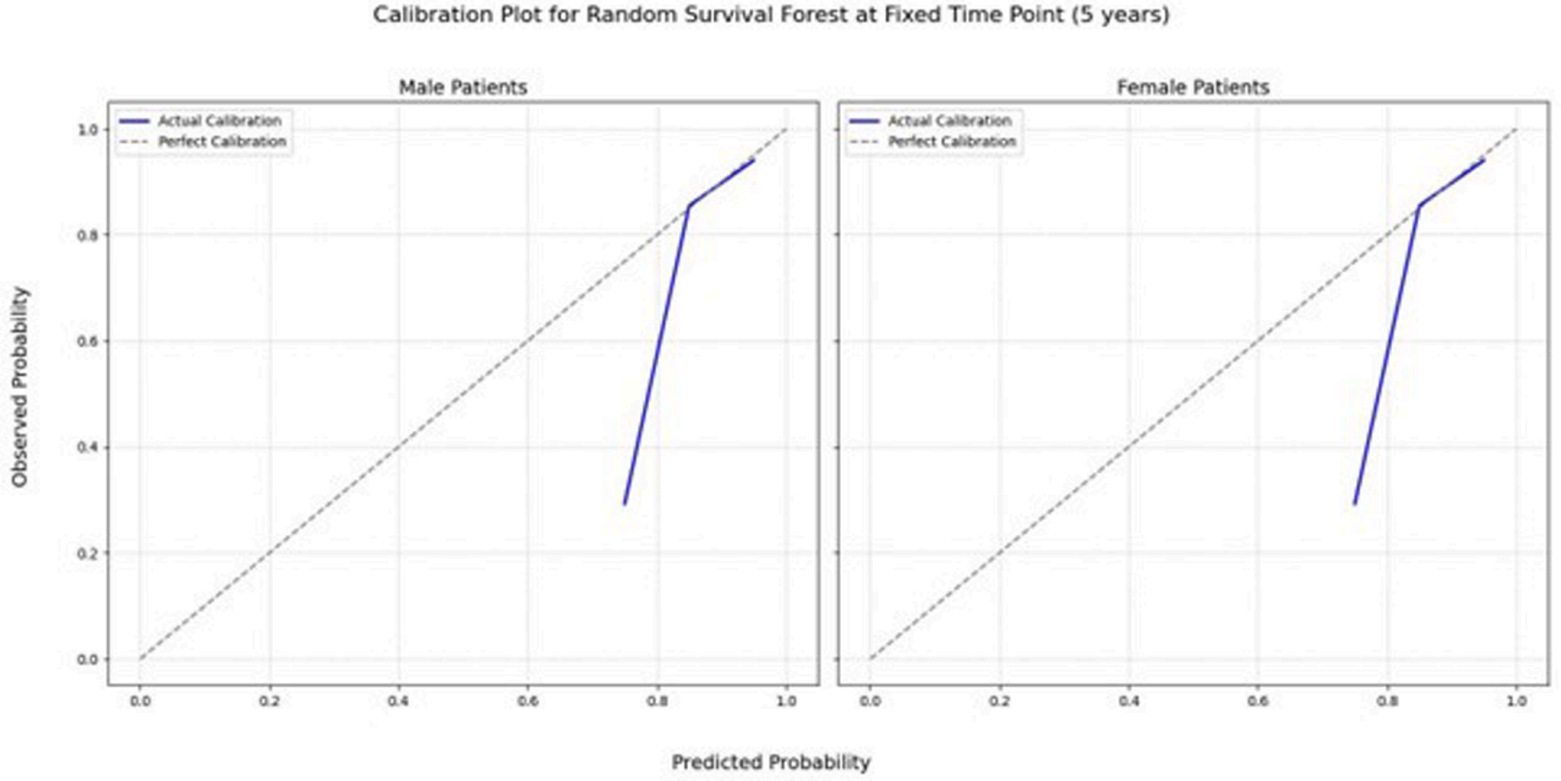
Calibration plot for death during follow-up.

**Figure E4:**
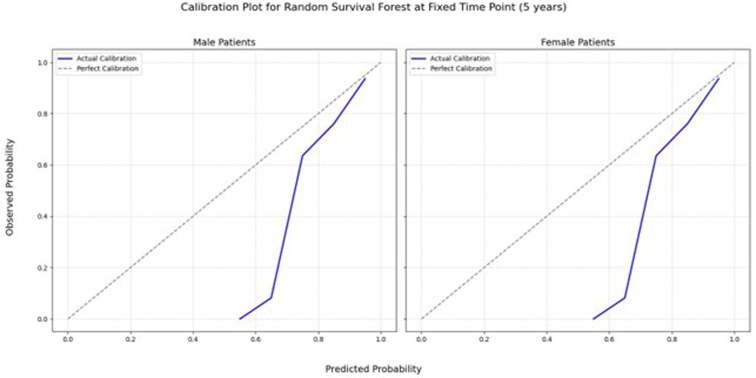
Calibration plot for revascularization.

Missing data were imputed with multiple imputation by chained equations (ie, MICE), and propensity score overlap weighting was applied (details in Appendix E1). The balance of the dataset can be found in Figure E1. Weighted Kaplan-Meier curves were used to analyze long-term composite outcomes, with a landmark analysis excluding events within 30 days to focus on long-term sex effects. Risk factors were evaluated via weighted Fine-Gray competing risks regression for men and women separately; sex interactions were tested with interaction terms. Information on the covariate selection process can be found in Appendix E1. Forest plots display sex-specific HRs and interactions. Random survival forests were used as an exploratory tool to assess and visualize sex-specific prognostic patterns and to validate the relative importance of risk factors identified in the regression models (details in Appendix E1). Information on modelling calibration can be found in Figure E2, Figure E3, Figure E4. Statistical significance was set at α = 0.05.

## Results

### Patients’ Baseline Characteristics

When we compared the BITA with the non-BITA subgroups, there were numerous significant differences contrasting the patients. The patients who received BITAs were younger (65.5 years vs 71 years; *P* < .001), had a lower rate of diabetes mellitus (30% vs 37%; *P* < .001), had lower New York Heart Association (NYHA) scores (*P* < .001), and a generally lower perioperative risk by European System for Cardiac Operative Risk Evaluation (EuroSCORE) II (1.04 vs 1.48; *P* < .001). In the overall cohort, 600 patients (15.5%) were female.

Of the 2010 total unweighted patients of this cohort who received BITAs, 1742 (86.6%) were male, and 268 (13.3%) were female. Women were significantly older than men at the time of surgery (median 68.0 vs 65.0 years, *P* < .001) and had greater EuroSCORE II values (median 1.40 vs 0.99, *P* < .001), reflecting a greater estimated perioperative risk. Smoking was notably less common among women (26% vs 44%, *P* < .001); likewise, the prevalence of dyslipidemia was lower in female patients (79% vs 85%, *P* = .031). A detailed account of all unweighted differences can be found in Table 1 and Table E1.

**Table 1:** Patient baseline characteristics

Characteristic	Overall BITA, N = 2010∗	Sex		*P* value†
		Female, n = 268∗	Male, n = 1742∗	
Age, y	65.50 (60.00, 71.00)	68.00 (62.50, 73.00)	65.00 (59.00, 71.00)	<.001
BMI	27.50 (25.20, 30.20)	27.15 (24.80, 30.25)	27.50 (25.30, 30.20)	.11
Diabetes mellitus	600 (30%)	87 (32%)	513 (29%)	.3
Dyslipidemia	1688 (84%)	213 (79%)	1475 (85%)	.031
History of hypertension	1664 (83%)	229 (85%)	1435 (82%)	.2
History of arrhythmia	129 (6.4%)	15 (5.6%)	114 (6.5%)	.6
History of smoking	837 (42%)	69 (26%)	768 (44%)	<.001
Peripheral artery disease	289 (14%)	32 (12%)	257 (15%)	.2
History of COPD	160 (8.0%)	15 (5.6%)	145 (8.3%)	.12
History of myocardial infarction	588 (29%)	75 (28%)	513 (29%)	.6
History of stroke	117 (5.8%)	19 (7.1%)	98 (5.6%)	.3
History of malignancy	30 (1.5%)	5 (1.9%)	25 (1.4%)	.6
NYHA				.036
1	319 (16%)	36 (13%)	283 (16%)	
2	989 (49%)	131 (49%)	858 (49%)	
3	674 (34%)	92 (34%)	582 (33%)	
4	28 (1.4%)	9 (3.4%)	19 (1.1%)	
Chronic renal failure	29 (1.4%)	4 (1.5%)	25 (1.4%)	>.9
EuroSCORE II	1.04 (0.75, 1.51)	1.40 (0.99, 2.04)	0.99 (0.73, 1.42)	<.001
Preoperative hemoglobin	14.50 (13.40, 15.30)	13.60 (12.50, 14.50)	14.60 (13.60, 15.40)	<.001
Preoperative thrombocyte count	221.00 (189.00, 262.00)	244.50 (204.00, 291.50)	219.00 (187.00, 257.00)	<.001
Preoperative CK-MB	14.00 (11.00, 18.00)	14.00 (11.00, 19.00)	14.00 (11.00, 18.00)	.4
Preoperative troponin	12.00 (10.00, 30.00)	10.00 (8.00, 26.80)	12.00 (10.00, 31.00)	.003
Preoperative leukocyte count	10 (1.3%)	2 (1.7%)	8 (1.2%)	.7
Preoperative LVEF <30%	956 (51%)	124 (48%)	832 (51%)	.4
Chronic total occlusion present				

*BITA*, Bilateral internal thoracic artery; *BMI*, body mass index; *COPD*, chronic obstructive pulmonary disease; *NYHA*, New York Heart Association; *EuroSCORE*, European System for Cardiac Operative Risk Evaluation; *CK-MB*, creatine kinase-MB; *LVEF*, left ventricular ejection fraction.

∗ Median (Q1, Q3); n (%).

† Wilcoxon rank sum test; Pearson χ^2^ test; Fisher exact test.

### Intraoperative Characteristics

In the overall cohort, patients who received BITAs had a greater number of bypasses performed (median 4 vs median 3; *P* < .001), a greater rate of complete revascularization (78% vs 70%; *P* < .001), and less stenosis in coronary segments 1 and 2 (both *P* < .001).

In the BITA subgroup, women had shorter cardiopulmonary bypass times (median 85 vs 88 minutes, *P* = .025) and aortic crossclamp times (68 vs 69 minutes, *P* = .031) compared with men. Graft diameters were consistently smaller in female patients, with both LITS and RITA diameters significantly lower than in male patients (LITA: 2.70 vs 2.90 mm and RITA: 2.70 vs 2.90 mm, both *P* < .001). Lastly, measured median flow of the LITA graft used in female patients was lower than in male patients (43 mL/min vs 60 mL/min, *P* = .007), whereas the RITA graft flow did not differ significantly (60 mL/min vs 60 mL/min, *P* = .200). Tables 2 and E2 show all investigated intraoperative characteristics.

**Table 2:** Intraoperative characteristics

Characteristic	Overall BITA, N = 2010∗	Sex		*P* value†
		Female, n = 268∗	Male, n = 1742∗	
No. of bypasses	4.00 (3.00, 4.00)	3.00 (3.00, 4.00)	4.00 (3.00, 4.00)	.2
Complete revascularization	1441 (78%)	201 (80%)	1240 (77%)	.300
No. of affected vessels	3 (3, 3)	3 (3, 3)	3 (3, 3)	.8
Time of cardiopulmonary bypass, min	87 (70, 104)	85 (68, 99)	88 (71, 105)	.025
Time of aortic crossclamping, min	69 (55, 85)	68 (53, 80)	69 (55, 85)	.031
Total time of surgery, min	240 (201, 262)	230 (200, 260)	240 (205, 262)	.12
LITA diameter, min	2.80 (2.50, 3.00)	2.70 (2.35, 3.00)	2.90 (2.50, 3.00)	<.001
RITA diameter, min	2.90 (2.50, 3.10)	2.70 (2.40, 3.00)	2.90 (2.50, 3.10)	<.001
Min. flow in LITA graft, mL/min	60 (40, 90)	43 (30, 83)	60 (40, 90)	.007
Min. flow in RITA graft, mL/min	60 (35, 88)	60 (35, 80)	60 (36, 90)	.2

*BITA*, Bilateral internal thoracic artery; *LITA*, left internal thoracic artery; *RITA*, right internal thoracic artery.

∗ Median (Q1, Q3); n (%).

† Wilcoxon rank sum test; Pearson χ^2^ test.

### Postoperative Outcomes

Postoperative outcomes were first compared unadjusted between men and women. Female patients experienced significantly greater 5-year mortality (9.0% vs 5.7%, *P* = .049) and nonsignificantly greater 5-year coronary reintervention rates (11.0% vs 7.8%, *P* = .089) compared with male patients in the unweighted cohort. The cumulative composite end point occurred more frequently in female patients at both 1 year (11% vs 6.4%, *P* = .004) and 5 years (18% vs 13%, *P* = .024). Within 30 days, mortality was greater in women than in men (2.2% vs 0.5%, *P* = .006), and the 30-day composite end point of death or repeat revascularization occurred in 5.6% of women versus 4.0% of men (*P* = .20). In Table 3 we have listed all relevant postoperative parameters. Deep sternal wound infection occurred more frequently in women than in men (5.3% vs 2.9%, *P* = .044).

**Table 3:** Postoperative outcomes

Characteristic	Overall BITA, N = 2010∗	Sex		*P* value†
		Female, n = 268∗	Male, n = 1742∗	
Overall mortality	330 (16%)	61 (23%)	269 (15%)	.003
30-d mortality	14 (0.7%)	6 (2.2%)	8 (0.5%)	.006
1-y mortality	44 (2.2%)	15 (5.2%)	30 (1.7%)	<.001
5-y mortality	124 (6.2%)	24 (9.0%)	100 (5.7%)	.040
Overall coronary reinterventions	222 (11%)	39 (15%)	183 (11%)	.049
Coronary reinterventions after 1 y	104 (5.2%)	19 (7.1%)	85 (4.9%)	.13
Cumulative composite end point after 30 d	84 (4.2%)	15 (5.6%)	69 (4.0%)	.2
Cumulative composite end point after 1 y	142 (7.1%)	30 (11%)	112 (6.4%)	.004
Cumulative composite end point after 5 y	272 (14%)	48 (18%)	224 (13%)	.024
Length of hospital stay, d	13.00 (10.00, 16.00)	14.00 (12.00, 19.00)	13.00 (10.00, 16.00)	<.001
Length of ICU stay, d	0.94 (0.86, 1.49)	0.97 (0.89, 2.01)	0.93 (0.85, 1.10)	.001
No. of erythrocyte transfusions	1.00 (0.00, 3.00)	3.00 (2.00, 4.00)	1.00 (0.00, 2.00)	<.001
No. of thrombocyte transfusions	0.00 (0.00, 0.00)	0.00 (0.00, 0.00)	0.00 (0.00, 0.00)	.6
No. of FFP	0.00 (0.00, 3.00)	0.00 (0.00, 3.00)	0.00 (0.00, 3.00)	<.001
Postoperative permanent pacemaker	26 (1.3%)	3 (1.1%)	23 (1.3%)	>.9
Postoperative dialysis	34 (1.7%)	9 (3.4%)	25 (1.4%)	.037
Deep sternal infection	64 (3.2%)	14 (5.3%)	50 (2.9%)	.044
Postoperative stroke	36 (1.8%)	6 (2.2%)	30 (1.7%)	.6
Postoperative atrial fibrillation	325 (16%)	52 (19%)	273 (16%)	.12
Postoperative max. CK-MB	46.00 (36.00, 60.00)	51.00 (39.00, 69.00)	46.00 (36.00, 59.00)	<.001
Postoperative max. troponin	561.00 (354.00, 992.00)	546.00 (347.00, 1050.00)	564.00 (355.00, 974.00)	.7
Postoperative LVEF <30%	19 (1.0%)	2 (0.8%)	17 (1.1%)	>.9

**Figure 1:**
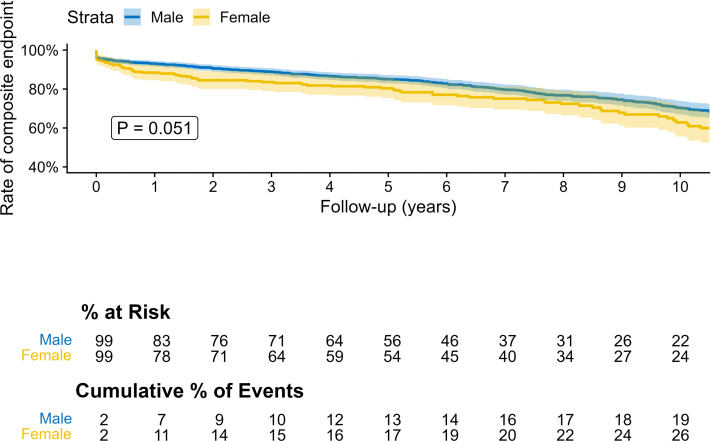
Weighted Kaplan-Meier survival curve of composite outcome of death and coronary revascularization during follow-up; *%*, percentage; *shaded areas* mark the 95% CIs.

**Figure 2:**
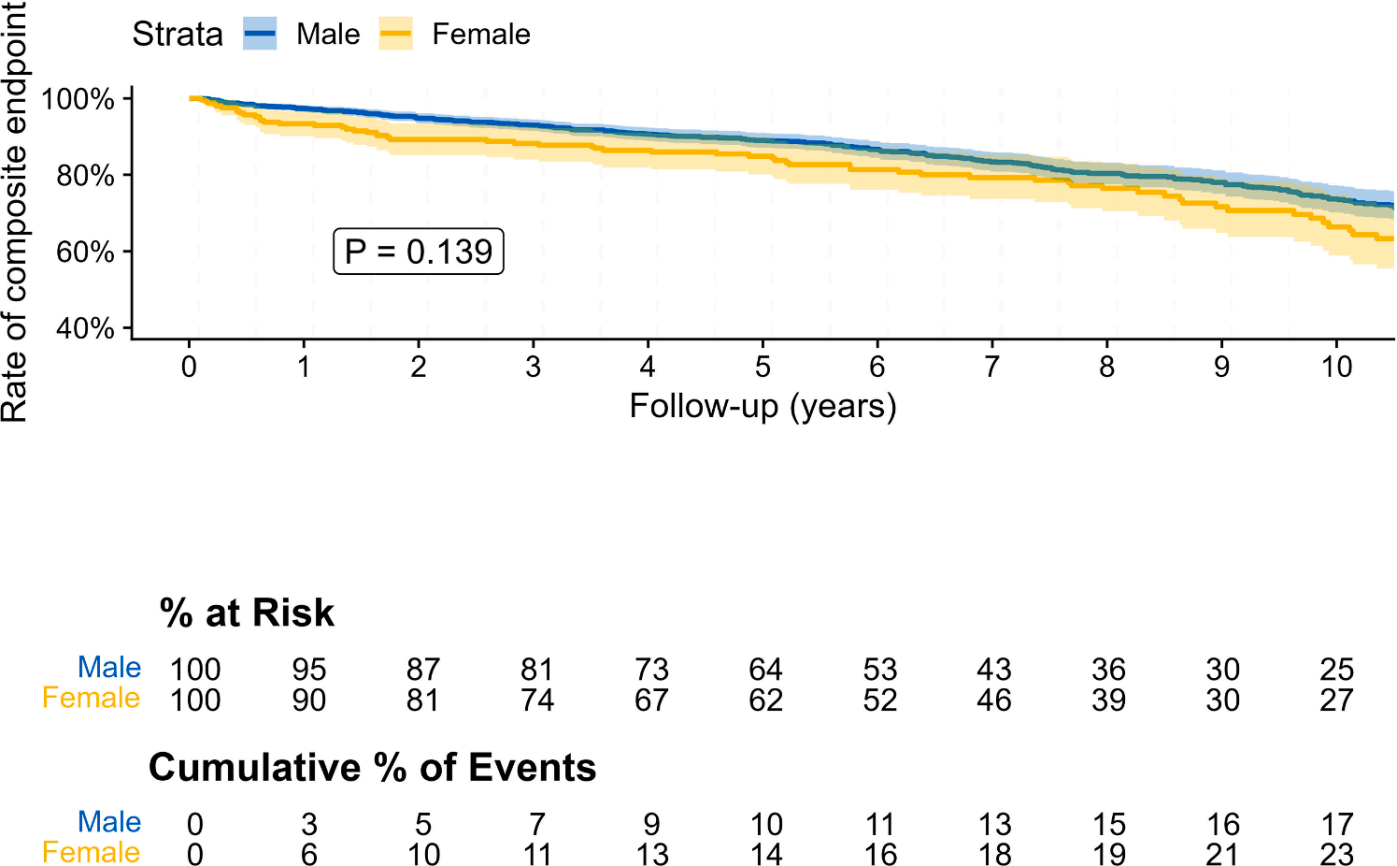
Weighted Kaplan-Meier survival curve of composite outcome of death and coronary revascularization during follow-up. Patients who had an outcome event within 30 days postoperatively were excluded; *%*, percentage; *shaded areas* mark the 95% CIs.

Values for 30-d, 1-y, and 5-y mortality and the composite end point are observed proportions at the specified time points; corresponding Kaplan-Meier curves with confidence intervals are shown in Figures 1 and 2.

*BITA*, Bilateral internal thoracic artery; *ICU*, intensive care unit; *FFP*, fresh-frozen plasma; *CK-MB*, creatine kinase-MB; *LVEF*, left ventricular ejection fraction.

∗ n (%); median (Q1, Q3).

† Pearson χ^2^ test; Wilcoxon rank sum test; Fisher exact test.

### Sex-Specific Outcomes (Main Analysis)

**Figure E5:**
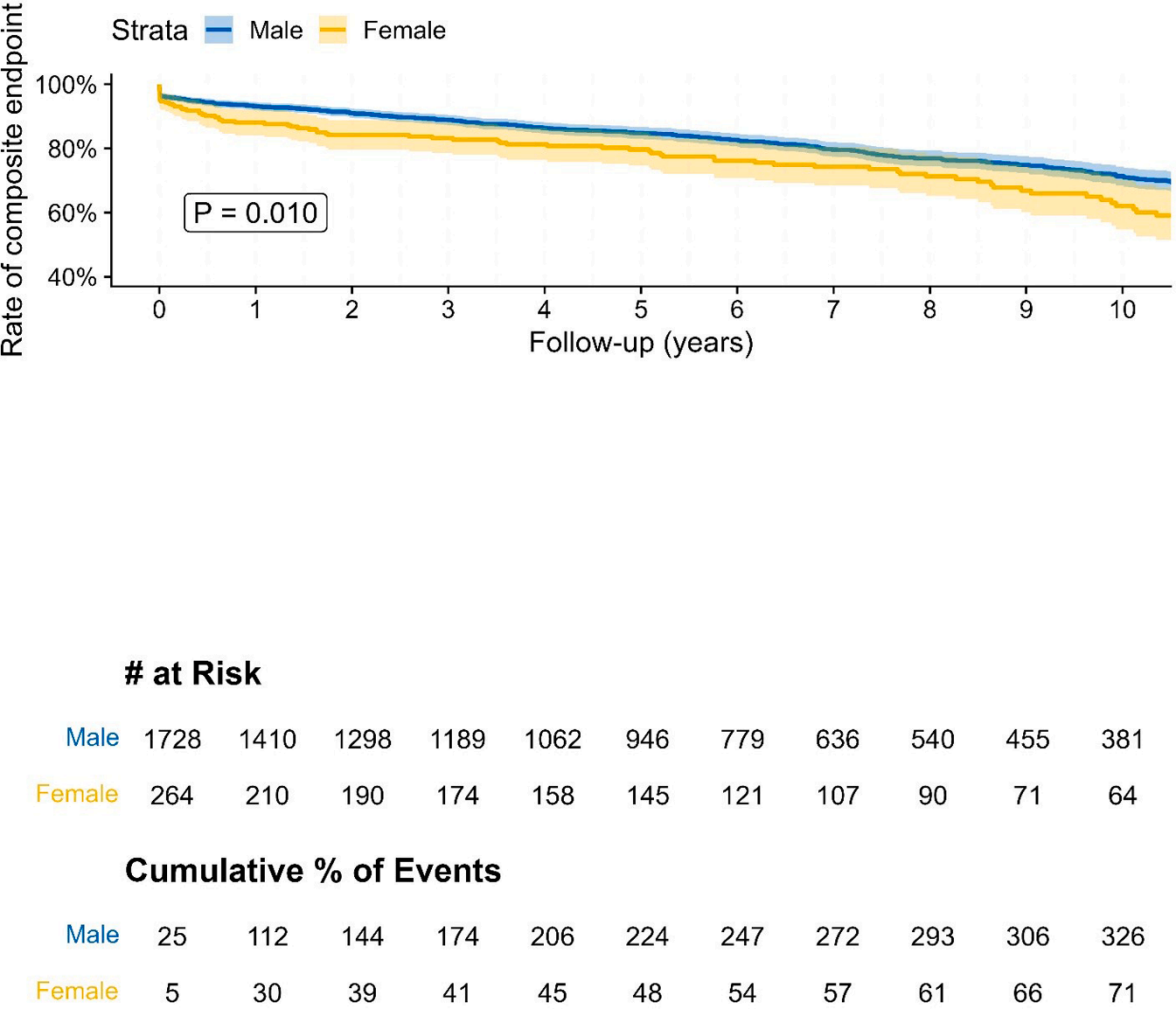
Unweighted Kaplan-Meier survival curve of the composite outcome of death and coronary revascularization during follow-up; %, percentage.

In weighted analyses accounting for baseline differences between sexes, the disparity in composite outcomes was substantially attenuated. Sex-specific outcomes after BITA-CABG revealed clear differences. In unweighted Kaplan-Meier analyses of the composite end point, female patients fared significantly worse than male patients, with a greater event rate throughout follow-up (*P* = .010) (Figure E5). When baseline differences were adjusted for further models, the outcome disparity diminished and did not reach statistical significance (*P* = .051) (Figure 1).

Exclusion of early (30-day) postoperative events further attenuated the sex-specific difference in risk, with the survival curves for men and women converging and the apparent difference no longer statistically significant (*P* = .139) (Figure 2). Interpretation of the results was facilitated in Table E3.

**Figure E6:**
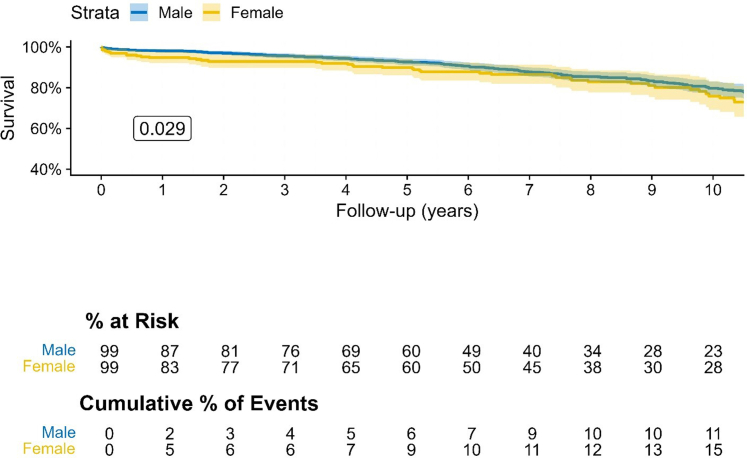
Weighted Kaplan-Meier survival curve of death during follow-up; %, percentage; the label annotates the *P* value; the *shaded areas* show the 95% CI.

**Figure E7:**
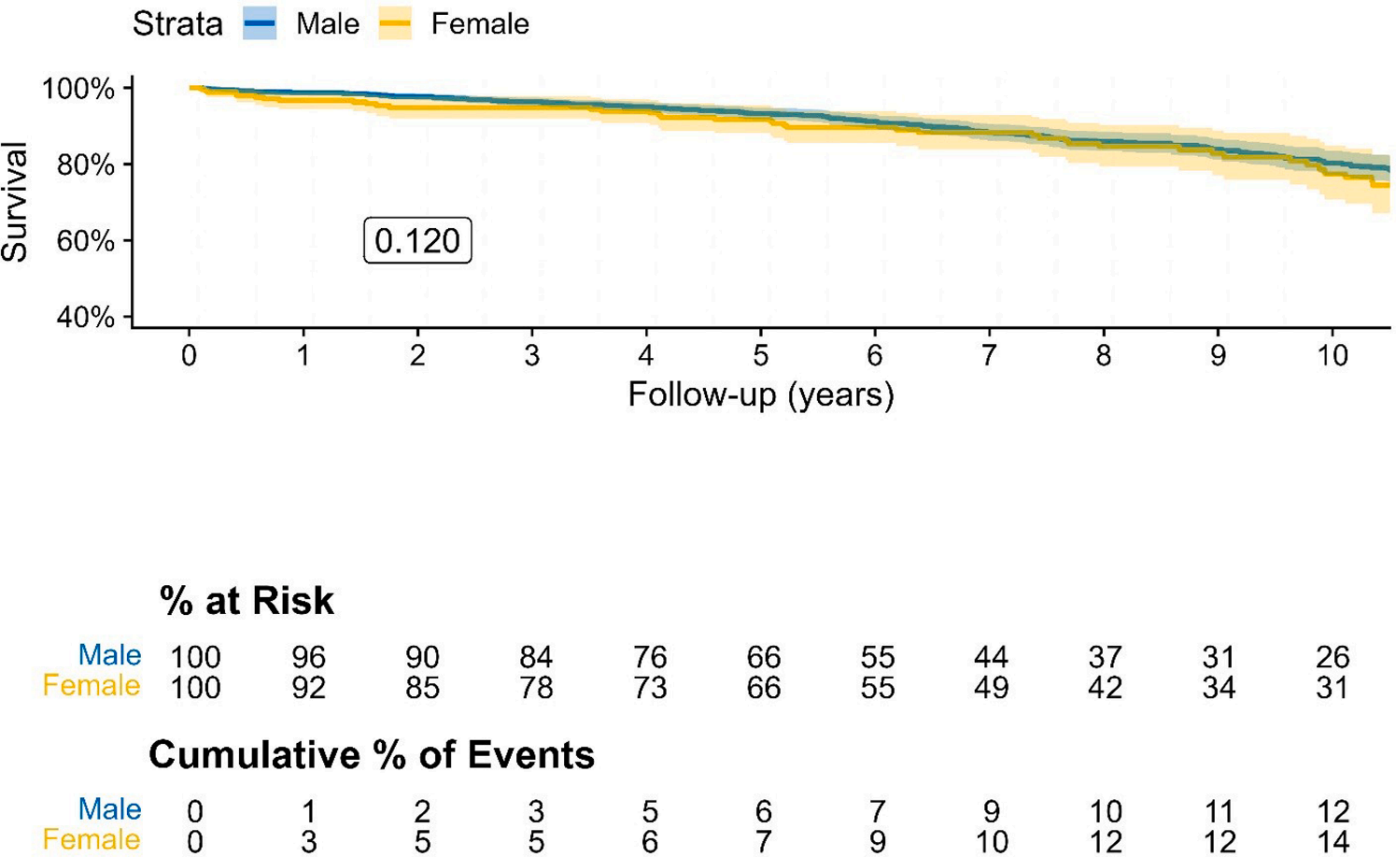
Weighted Kaplan-Meier survival curve of death during follow-up. Patients who had an outcome event within 30 days postoperatively were excluded; %, percentage; the label annotates the *P* value; the *shaded areas* show the 95% CI.

**Figure E8:**
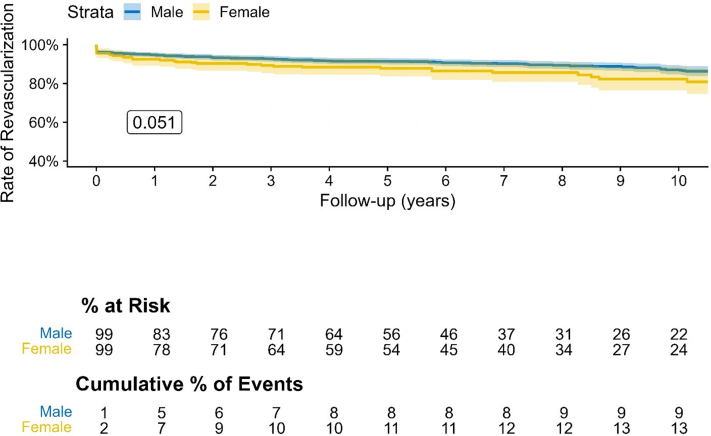
Weighted Kaplan-Meier survival curve of coronary revascularization during follow-up; %, percentage; the label annotates the *P* value; the *shaded areas* show the 95% CI.

**Figure E9:**
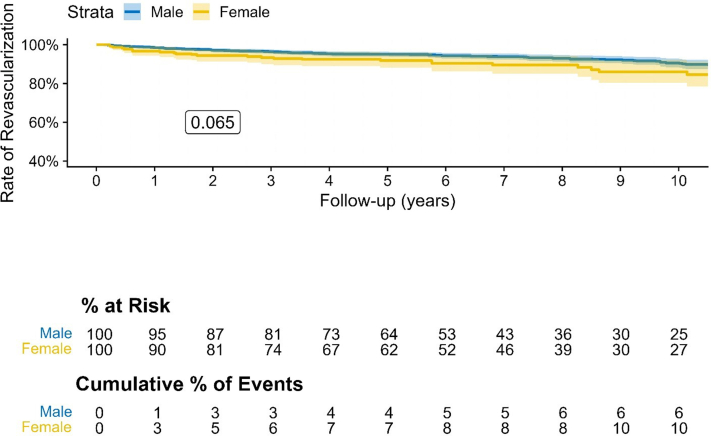
Weighted Kaplan-Meier survival curve of coronary revascularization during follow-up. Patients who had an outcome within 30 days postoperatively were excluded; %, percentage; the label annotates the *P* value; the *shaded areas* show the 95% CI.

**Figure E10:**
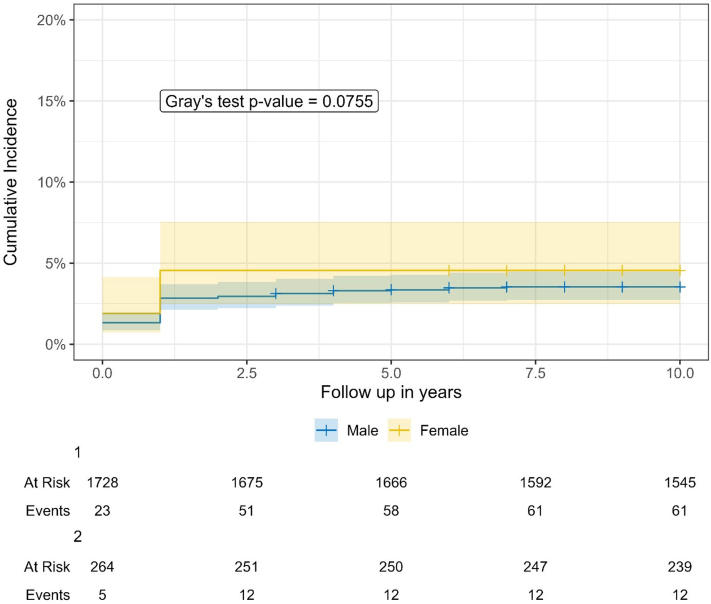
Unweighted cumulative incidence plot for coronary revascularization with the competing risk of death; the *shaded areas* show the 95% CI.

Figure E6, Figure E7, Figure E8, Figure E9, Figure E10 further include all unweighted Kaplan-Meier curves for total events, death during follow-up, and coronary revascularization, as well as the unweighted competing risk cumulative incidence plot.

### Different Effect of Risk Factors

**Figure 3:**
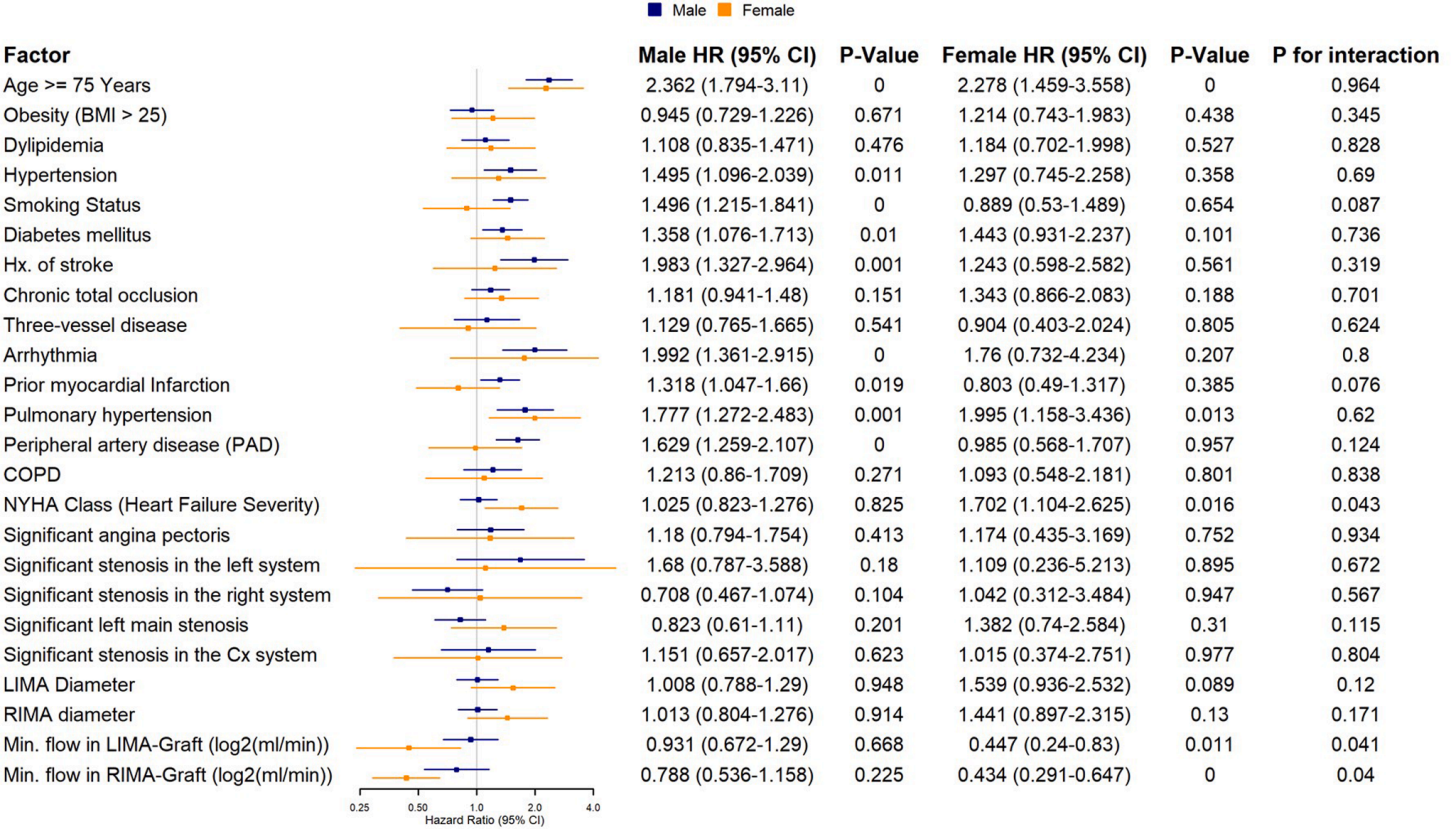
Forest plot showing the multivariable regression results for male and female patients regarding the composite outcome, as well as the interaction *P* value between the sexes. *HR*, Hazard ratio; *BMI*, body mass index; *COPD*, chronic obstructive pulmonary disease; *NYHA*, New York Heart Association; *LIMA*, left internal mammary artery/same as left internal thoracic artery; *RIMA*, right internal mammary artery/same as right internal thoracic artery.

Comprehensive risk factor analyses demonstrated distinct sex-specific patterns in their effects on long-term outcomes after BITA-CABG. Forest plots of interaction effects revealed that certain clinical features had dissimilar impacts in male and female patients (Figure 3). For women, greater NYHA class (hazard ratio [HR], 1.70; CI, 1.10-2.62), pulmonary hypertension (HR, 2.00; CI, 1.16-3.44), LITA graft flow (HR, 0.45; CI, 0.24-0.83), and RITA graft flow (HR, 0.43; CI, 0.29-0.65) were the strongest predictors of composite outcome, showing weaker effect in men. Interaction analysis revealed, that of these factors, only NYHA class and both graft flow rates showed statistically significant interaction (*P* < .05). In contrast, for men, smoking status (HR, 1.50; CI, 1.22-1.84), previous myocardial infarction (MI) (HR, 1.32; CI, 1.04-1.66), and peripheral artery disease (HR, 1.63; CI, 1.26-2.11) emerged as dominant contributors to greater risk. Previous MI and smoking status both showed statistically nonsignificant trends (*P* = .076, *P* = .087) regarding the interaction term.

**Figure 4:**
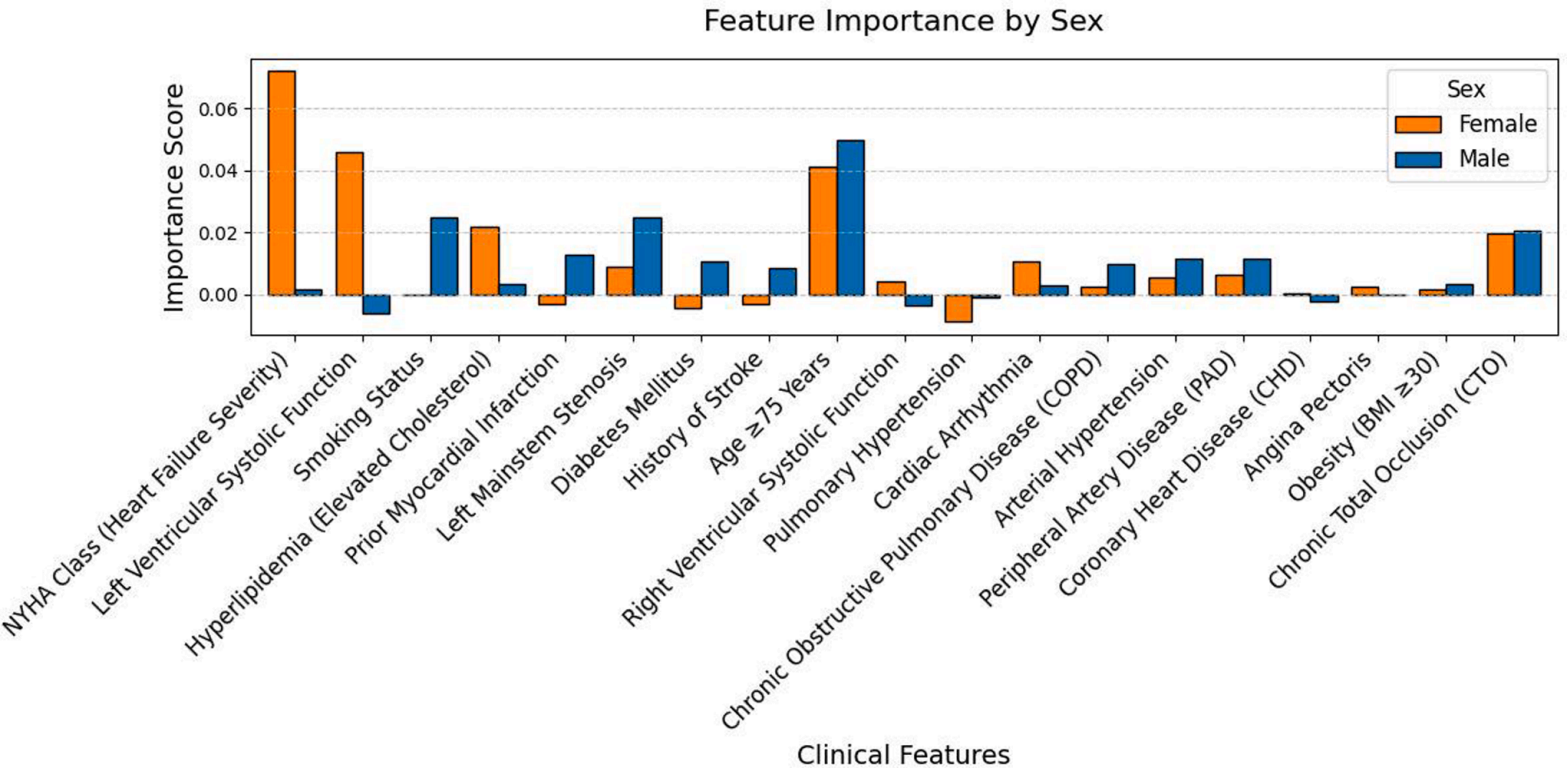
Bar chart showing the relative permutation importance score of investigated parameters of the trained random survival forest. *NYHA*, New York Heart Association; *BMI*, body mass index.

**Figure E11:**
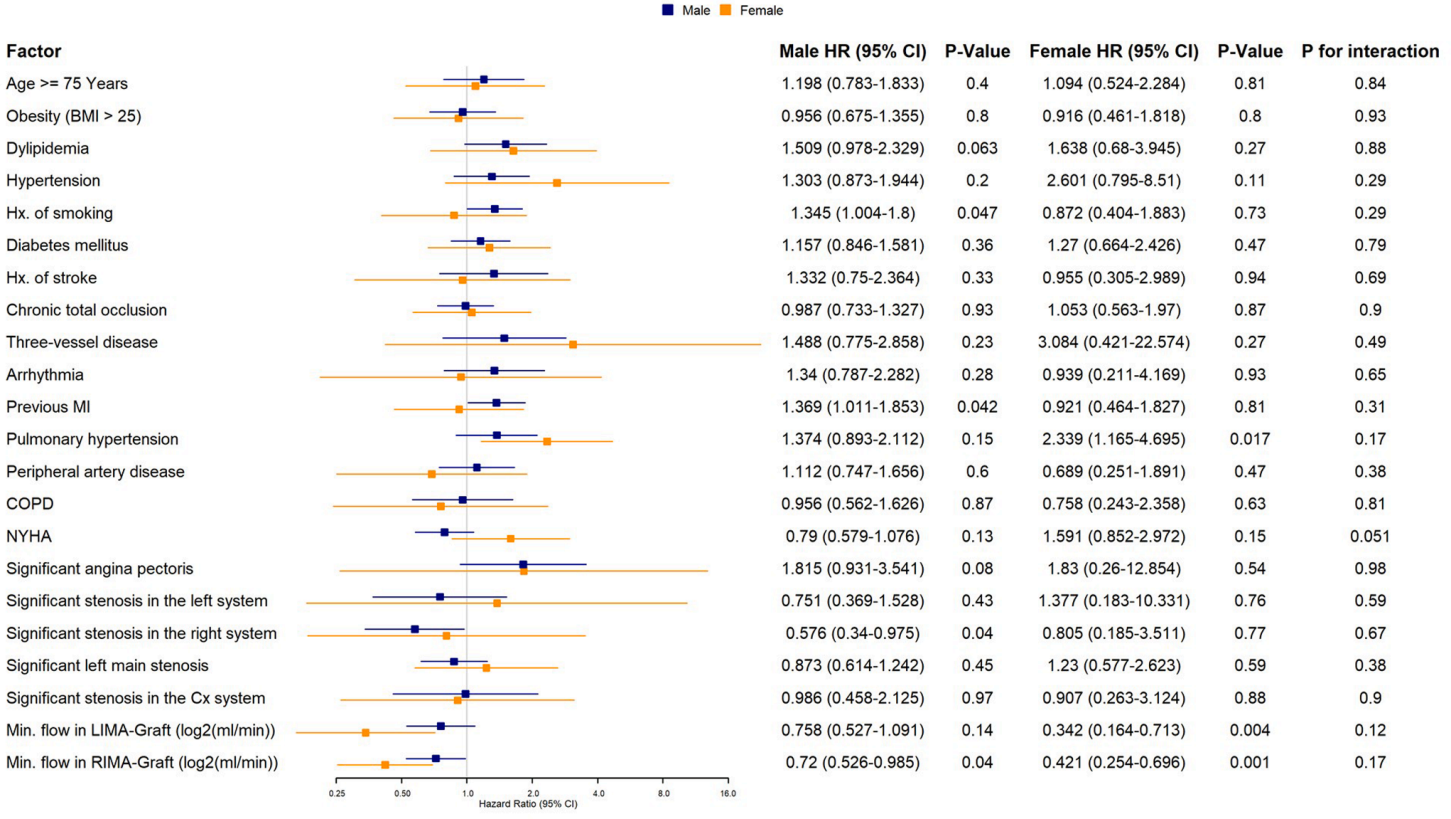
Forest plot showing the multivariable regression results for male and female patients regarding revascularization, as well as the interaction *P* value between the sexes. *BMI*, Body mass index; *Hx*., history; *MI*, myocardial infarction; *COPD*, chronic obstructive pulmonary disease; *NYHA*, New York Heart Association; *LIMA*, left internal mammary artery/same as left internal thoracic artery; *RIMA*, right internal mammary artery/same as right internal thoracic artery.

**Figure E12:**
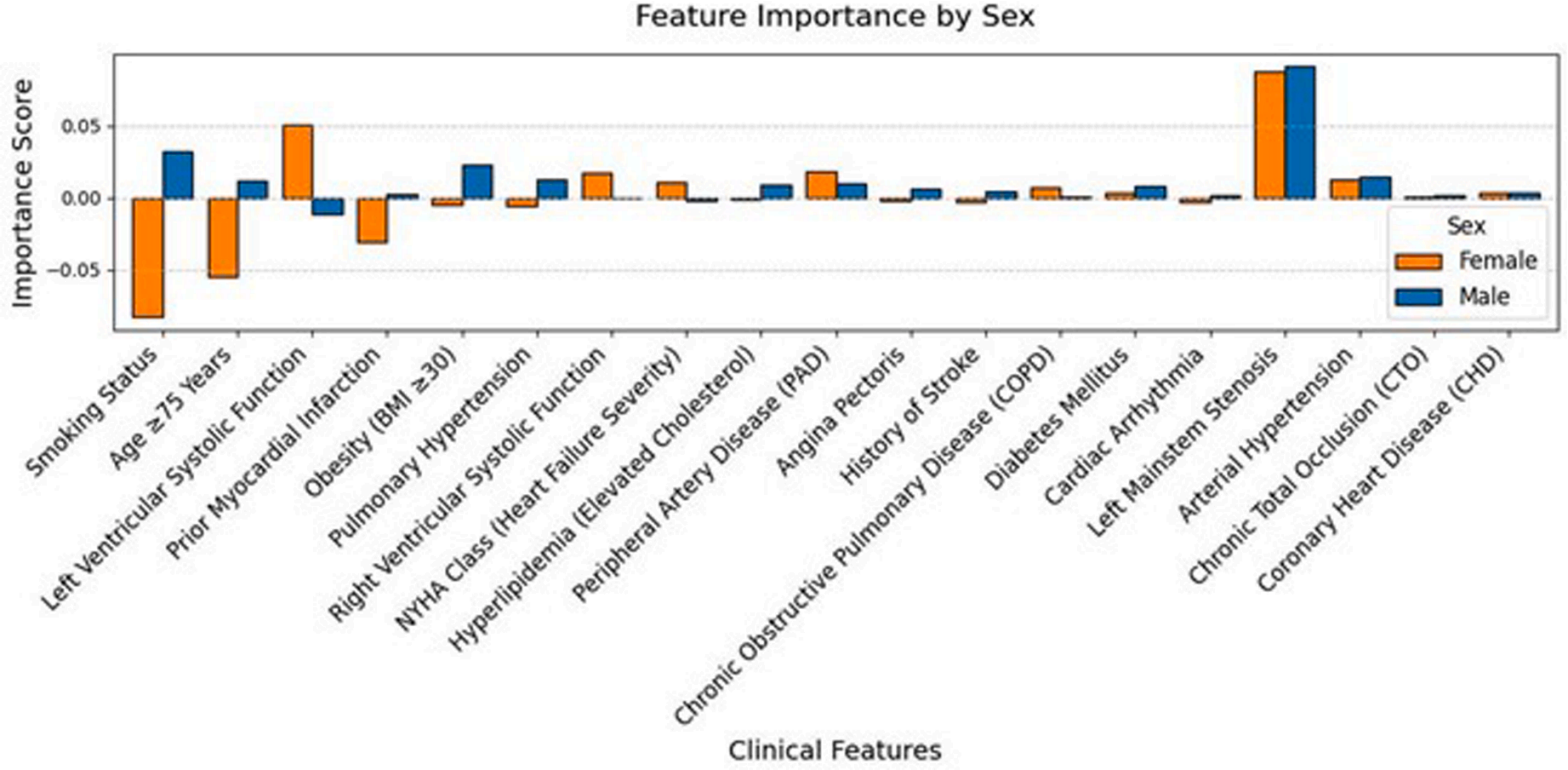
Bar chart showing the relative permutation importance score of investigated parameters of the trained random survival forest for predicting revascularization. *BMI*, Body mass index; *NYHA*, New York Heart Association.

**Figure E13:**
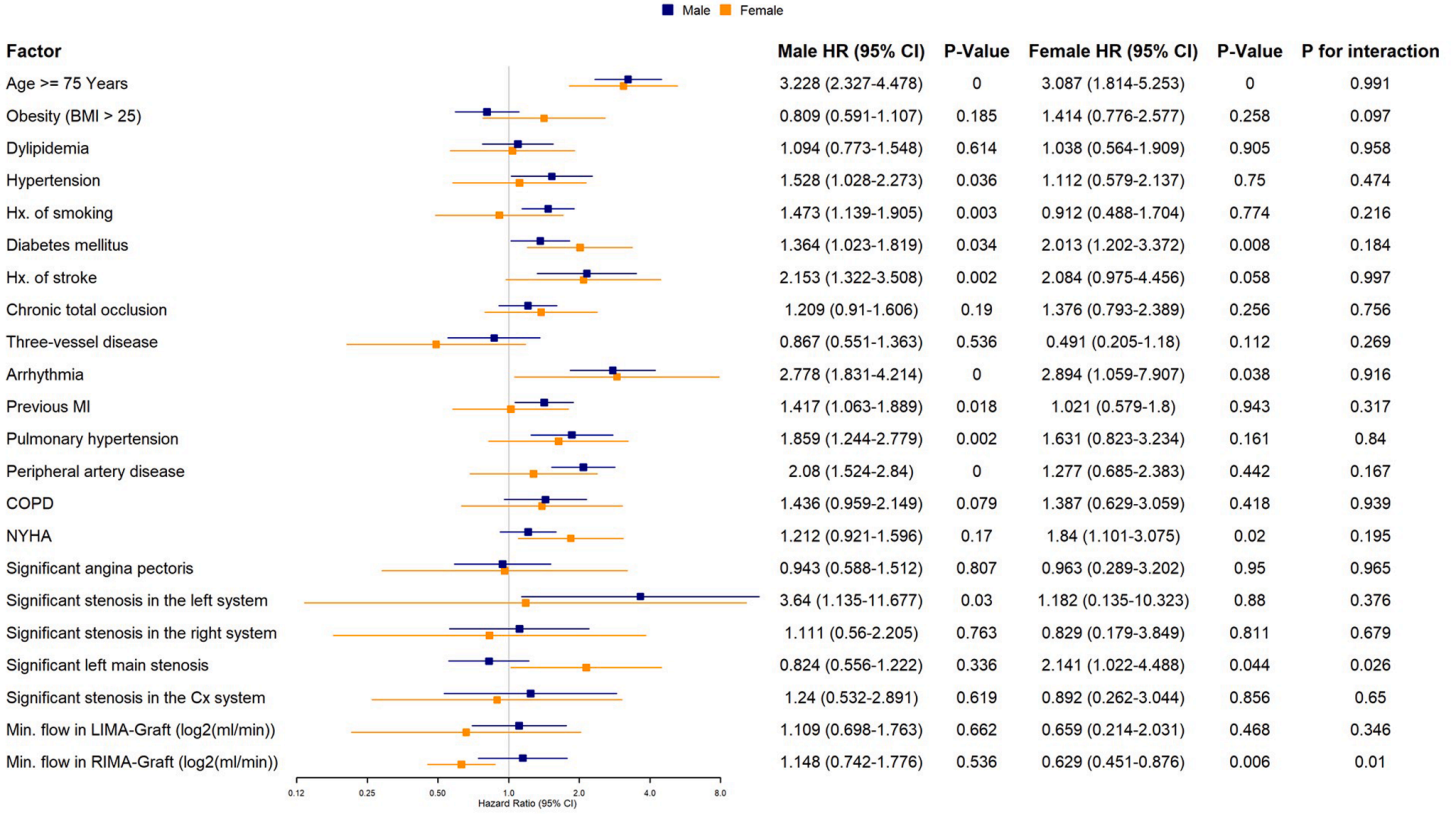
Forest plot showing the multivariable regression results for male and female patients regarding death, as well as the interaction *P* value between the sexes. *BMI*, Body mass index; *Hx*., history; *MI*, myocardial infarction; *COPD*, chronic obstructive pulmonary disease; *NYHA*, New York Heart Association; *LIMA*, left internal mammary artery/same as left internal thoracic artery; *RIMA*, right internal mammary artery/same as right internal thoracic artery.

**Figure E14:**
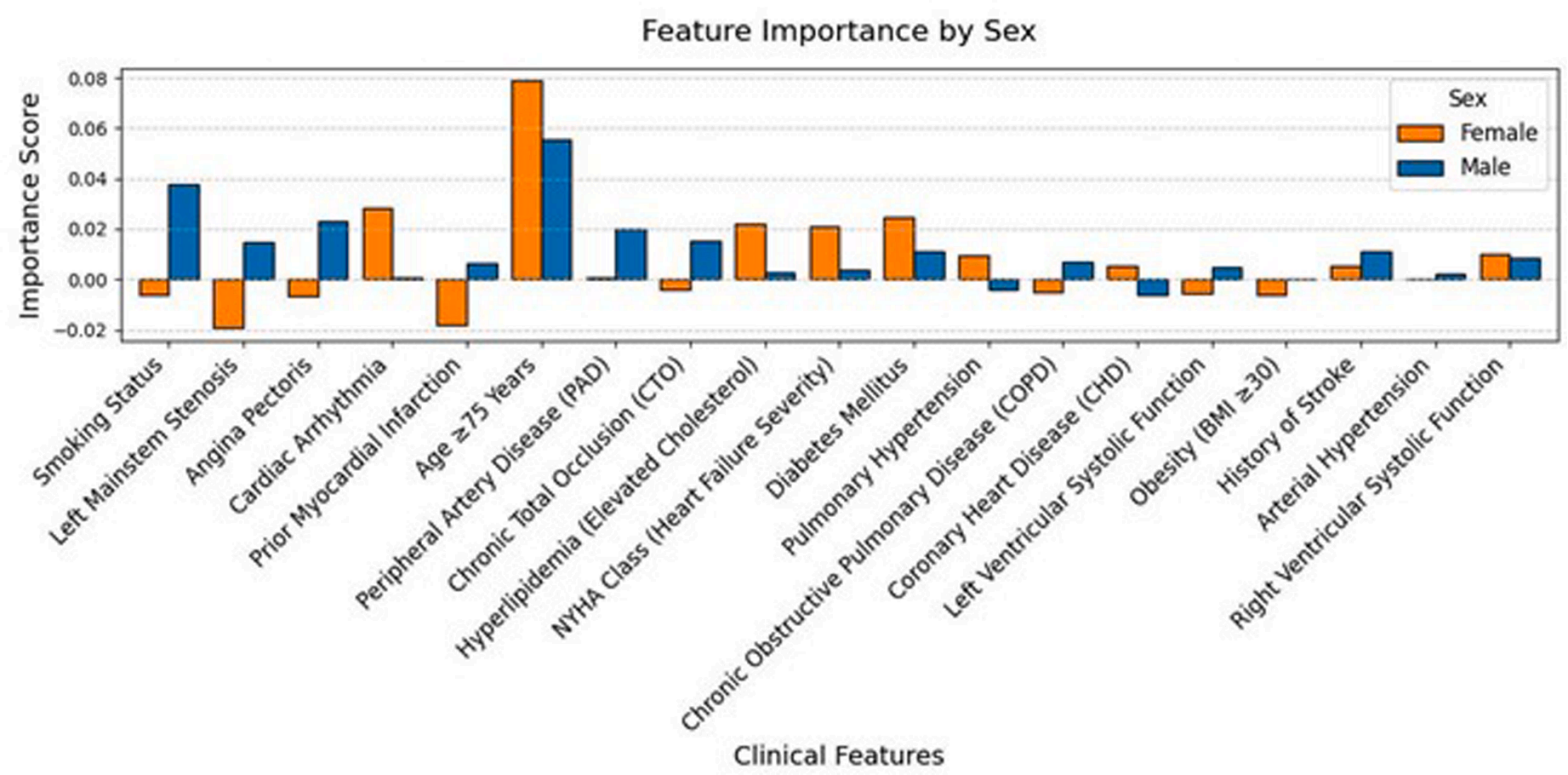
Bar chart showing the relative permutation importance score of investigated parameters of the trained random survival forest for predicting death. *NYHA*, New York Heart Association; *BMI*, body mass index.

Permutation-importance analysis from machine learning models further confirmed these findings. For example, NYHA class, pulmonary hypertension and LITA and RITA graft flow were among the topmost important predictors in female patients, whereas smoking and previous MI were more strongly predictive in males. Figure 4 illustrates these relative importance scores derived from the random survival forest. Note that both large negative and positive scores would denote importance. The Figures illustrating the interactions and results of the random survival forest for the endpoints revascularization and death can be found in the supplemental Figure E11, Figure E12, Figure E13, Figure E14.

## Discussion

In this study, 3 major findings emerged regarding sex differences in outcome after BITA-CABG: (1) Sex-based disparities in long-term outcomes are substantially reduced after adjustment for baseline characteristics, yet women experienced significantly greater risk for early postoperative events. (2) After the initial 30-day period, there were no persistent long-term sex differences in composite end-point rates. (3) Analyses identified distinct risk profiles for adverse outcomes highlighting disparity between sexes in relevant predictors.

The central and novel observation of this study is that sex-based disparities in outcomes after BITA-CABG are largely confined to the early postoperative period: after overlap weighting and exclusion of 30-day events, long-term mortality and repeat revascularization appeared broadly similar between the sexes. In addition, distinct risk profiles were present, with heart failure severity, pulmonary hypertension, and graft flow being most relevant in women and atherothrombotic factors such as smoking, previous infarction, and peripheral artery disease dominating in men.

### Baseline Characteristics

Generally speaking, the patients who received BITAs differed significantly from the non-BITA cohort in several key factors affecting baseline perioperative risk. As a result of these differences, as well as the assumed benefits of BITA surgery, which may mask the sex disparities as the result of better long-term patency and lower graft-vessel mismatch, isolated analysis of sex differences in patients who receive BITAs seems necessary.^12^^,^^13^ Baseline and intraoperative characteristics showed several notable sex-specific differences in the patients who received BITAs. Women had significantly greater perioperative risk, as reflected by EuroSCORE II. The lower prevalence of smoking and dyslipidemia among women aligns with previous large-scale studies showing that female patients who undergo CABG present at older ages and with distinct cardiovascular risk profiles.^14^, ^15^, ^16^ NYHA class IV was more common in women, showing more severely symptomatic heart failure. Most other risk factors were similar between sexes. Thus, female patients undergoing BITA-CABG differ mainly in perioperative risk, not in surgical approach or disease extent, highlighting the need for individualized perioperative management and attention to risk factors that disproportionately affect women. In addition, the 30-day cumulative composite end point (4.2%-5.6%) appears relatively high but reflects a broad definition that combines all-cause mortality with both surgical and percutaneous reinterventions in a real-world cohort of older, comorbid patients with complex multivessel disease.

### Outcomes Improve After Adjusting for Baseline Differences

Female patients had more events after BITA-CABG in unadjusted analyses, but this was largely reduced after adjusting for patient and surgical factors. Removing early (30-day) events eliminated sex differences in survival, showing that the increase excess risk for women is concentrated in the immediate postoperative period, consistent across weighted and unweighted analyses.

These findings align with previous studies, which identify early postoperative mortality and adverse events as being significantly greater in women undergoing CABG, with differences diminishing or becoming nonsignificant when adequate adjustment for baseline patient characteristics is applied.^8^^,^^17^, ^18^, ^19^ In particular, female sex is an established independent risk factor for short-term morbidity and mortality after cardiac surgery, frequently attributed to advanced age at presentation, greater comorbidity burden, smaller vessel size, and perioperative anemia.^16^ Several robust studies have shown that in non-BITA cohorts, after propensity matching or risk adjustment, outcomes for women are similar to men beyond the initial postoperative phase, and the persistent sex difference is largely explained by early events.^17^

The greater incidence of early adverse events among women after BITA-CABG appears largely attributable to anatomical and physiological differences that intensify perioperative risk. Specifically, smaller coronary and conduit vessel diameters in female patients increase technical complexity and the likelihood of early graft occlusion, MI, and repeat revascularization. This vulnerability is further compounded by older age, greater frailty, and greater rates of perioperative anemia, which heighten susceptibility to bleeding, infection, and organ dysfunction.^20^ Importantly, these factors converge in the immediate postoperative period and account for most of the excess risk observed in women. However, once female patients overcome this critical phase and perioperative parameters stabilize, subsequent outcomes align closely with those of men, suggesting that acute procedural and physiological factors, rather than ongoing sex disparities, primarily drive early postoperative risk in women after CABG.

### Distinct Risk Factor Profiles Between the Sexes

Risk factor analysis identified different long-term predictors for men and women after BITA-CABG. In women, more symptomatic heart failure and pulmonary hypertension were stronger predictors of adverse outcomes, and low graft flow was associated with early events only in women. These results underscore key sex-specific risks and the importance of tailored management after surgery.

By contrast, in men, traditional risk factors, that is, smoking status, previous MI, and peripheral artery disease, were the main drivers of risk, with weaker relevance to outcomes in women. These findings mirror those of previous studies in cardiovascular disease populations, which reveal sex differences in both the prevalence and the prognostic importance of cardiovascular risk factors: NYHA class and pulmonary hypertension are well documented to be more prevalent and hazardous in women,^21^ whereas smoking and previous MI consistently exert greater long-term impact in men.^22^^,^^23^

These distinct risk factor profiles, supported by machine learning permutation importance, indicate that late adverse events after CABG are driven by fundamentally different mechanisms in women and men. In women, prognosis is more strongly influenced by heart failure symptom severity and pulmonary hypertension, whereas in men, atherothrombotic factors such as smoking and previous infarction predominate. These findings underscore the need for sex-specific monitoring and targeted therapies after BITA-CABG. In addition, lower graft flow in women, likely related to smaller vessel and conduit size, might increase risk for early graft failure and death by promoting resistance, turbulence, vasospasm, and thrombosis; these factors may amplify the impact of suboptimal flow by promoting acute endothelial injury, platelet activation, and prothrombotic cascades, ultimately leading to graft occlusion and adverse clinical outcomes.^24^^,^^25^ This suggests that intraoperative flow targets may need to be tailored by sex to improve outcomes.

### Limitations and Strengths

The main limitation of this study is its retrospective, single-center design, which can lead to confounding and limits how broadly the results apply. Unmeasured factors may have influenced outcomes, especially in subgroups like women. Strengths include a large modern cohort, detailed risk stratification, and advanced analytic methods, offering important new insights into sex-specific risks in BITA-CABG. Moreover, the sex-stratified random survival forest models showed only moderate discrimination and suboptimal calibration, likely reflecting the limited number of events relative to model complexity; as such, their results should be considered exploratory and complementary to other analyses.

Radial artery grafting is very rarely used at our institution, which precluded meaningful comparisons of BITA versus alternative second conduits in women; such comparisons are an important focus for future research. Importantly, our analyses were restricted to patients selected for BITA CABG and did not include patients treated with alternative conduit strategies or PCI. Therefore, our study does not establish whether BITA grafting is advantageous, neutral, or detrimental for women compared with other revascularization approaches. In addition, because cause-specific mortality data were not consistently available, we relied on all-cause death and could not distinguish cardiac from noncardiac deaths, which limits mechanistic interpretation of the observed sex differences. Lastly, our analysis is restricted to patients selected for BITA-CABG at a single tertiary center and does not include individuals with coronary artery disease who received other forms of revascularization or no surgery; therefore, the generalizability of our findings to the broader population of men and women with coronary artery disease is limited.

## Conclusions

Sex-specific differences after BITA-CABG were most pronounced in the early postoperative period, with women experiencing greater risk of death or repeat revascularization. After adjustment for baseline differences and exclusion of early events, long-term outcomes appeared broadly similar between men and women, although residual confounding and selection of lower-risk women for BITA cannot be excluded. Heart failure symptom severity, pulmonary hypertension, and graft flow are key risks for women, whereas smoking, previous MI, and peripheral artery disease drive outcomes in men. These findings highlight that even with arterial revascularization using BITA, sex-specific management remains essential, with particular emphasis in women on optimizing heart failure symptoms, pulmonary hypertension, and graft flow.

### Declaration of Generative AI and AI-Assisted Technologies in the Writing Process generative-AI

During the preparation of this work, the authors used Grammarly in order to perform spell-checking and grammatical amplification. After using this tool/service, the authors reviewed and edited the content as needed and take full responsibility for the content of the publication.

## Conflict of Interest Statement

M.C. reported consultancy fees from Medira, NEOS, Terumo Aortic, Medtronic, and Endospan; a speaking honorarium from Abbott; payment to their institution from Terumo Aortic for postmarket registries for study nurses; and shares from TEVAR Ltd. and Ascense Medical. M.W. reported speaker fees/honoraria from Edwards Lifesciences, Abbott Vascular, IPPMed, AstraZeneca, and Philips and travel expenses from Bayer. All other authors reported no conflicts of interest.

The *Journal* policy requires editors and reviewers to disclose conflicts of interest and to decline handling or reviewing manuscripts for which they may have a conflict of interest. The editors and reviewers of this article have no conflicts of interest.

## Figures and Tables

**Table E1:** Patient baseline characteristics together with the overall cohort

Characteristic	Overall, N = 3867∗	BITA		*P* value†	Overall BITA, n = 2010∗	Sex		*P* value†
		BITA, n = 20,10	Non-BITA, n = 18,57			Female, n = 268	Male, n = 1742∗	
Age, y	68.00 (61.00, 74.00)	65.50 (60.00, 71.00)	71.00 (64.00, 76.00)	<.001	65.50 (60.00, 71.00)	68.00 (62.50, 73.00)	65.00 (59.00, 71.00)	<.001
BMI	27.40 (25.10, 30.10)	27.55 (25.30, 30.20)	27.30 (24.90, 30.10)	.2	27.50 (25.20, 30.20)	27.15 (24.80, 30.25)	27.50 (25.30, 30.20)	.11
Diabetes mellitus	1250 (33%)	591 (30%)	659 (37%)	<.001	600 (30%)	87 (32%)	513 (29%)	.3
Dyslipidemia	2964 (82%)	1606 (84%)	1358 (79%)	<.001	1688 (84%)	213 (79%)	1475 (85%)	.031
History of hypertension	3050 (84%)	1594 (83%)	1456 (84%)	.2	1664 (83%)	229 (85%)	1435 (82%)	.2
History of arrhythmia	310 (8.0%)	129 (6.4%)	181 (9.8%)	<.001	129 (6.4%)	15 (5.6%)	114 (6.5%)	.6
History of smoking	1528 (40%)	833 (42%)	695 (38%)	.012	837 (42%)	69 (26%)	768 (44%)	<.001
Peripheral artery disease	500 (15%)	242 (14%)	258 (16%)	.034	289 (14%)	32 (12%)	257 (15%)	.2
History of COPD	360 (9.3%)	160 (8.0%)	200 (11%)	.002	160 (8.0%)	15 (5.6%)	145 (8.3%)	.12
History of myocardial infarction	1296 (34%)	585 (29%)	711 (39%)	<.001	588 (29%)	75 (28%)	513 (29%)	.6
History of stroke	233 (6.0%)	117 (5.8%)	116 (6.3%)	.6	117 (5.8%)	19 (7.1%)	98 (5.6%)	.3
History of malignancy	59 (1.9%)	25 (1.5%)	34 (2.3%)	.11	30 (1.5%)	5 (1.9%)	25 (1.4%)	.6
NYHA				<.001				.036
1	493 (14%)	295 (16%)	198 (12%)		319 (16%)	36 (13%)	283 (16%)	
2	1667 (47%)	916 (49%)	751 (45%)		989 (49%)	131 (49%)	858 (49%)	
3	1274 (36%)	614 (33%)	660 (39%)		674 (34%)	92 (34%)	582 (33%)	
4	88 (2.5%)	26 (1.4%)	62 (3.7%)		28 (1.4%)	9 (3.4%)	19 (1.1%)	
Chronic renal failure	99 (2.6%)	29 (1.5%)	70 (3.9%)	<.001	29 (1.4%)	4 (1.5%)	25 (1.4%)	>.9
EuroSCORE II	1.23 (0.85, 1.92)	1.04 (0.75, 1.51)	1.48 (0.99, 2.35)	<.001	1.04 (0.75, 1.51)	1.40 (0.99, 2.04)	0.99 (0.73, 1.42)	<.001
Preoperative hemoglobin	14.30 (13.20, 15.20)	14.50 (13.40, 15.30)	14.20 (13.00, 15.10)	<.001	14.50 (13.40, 15.30)	13.60 (12.50, 14.50)	14.60 (13.60, 15.40)	<.001
Preoperative thrombocyte count	221.00 (187.00, 262.00)	221.00 (189.00, 262.00)	221.00 (185.00, 264.00)	.6	221.00 (189.00, 262.00)	244.50 (204.00, 291.50)	219.00 (187.00, 257.00)	<.001
Preoperative CK-MB	14.00 (11.00, 19.00)	14.00 (11.00, 18.00)	15.00 (11.00, 20.00)	<.001	14.00 (11.00, 18.00)	14.00 (11.00, 19.00)	14.00 (11.00, 18.00)	.4
Preoperative troponin	14.00 (10.00, 45.00)	12.00 (10.00, 30.00)	17.00 (10.00, 70.00)	<.001	12.00 (10.00, 30.00)	10.00 (8.00, 26.80)	12.00 (10.00, 31.00)	.003
Preoperative leukocyte count	7435.00 (6210.00, 9020.00)	7420.00 (6200.00, 8940.00)	7460.00 (6230.00, 9100.00)	.2	10 (1.3%)	2 (1.7%)	8 (1.2%)	.7
Preoperative LVEF <30%	63 (3.6%)	10 (1.3%)	53 (5.5%)	<.001	956 (51%)	124 (48%)	832 (51%)	.4
Chronic total occlusion present	1919 (52%)	956 (51%)	963 (54%)	.026				
Urgent (within 48 h)	310 (8.6%)	90 (4.9%)	220 (12%)	<.001	90 (4.9%)	15 (6.0%)	75 (4.7%)	.4

*BITA*, Bilateral internal thoracic artery; *BMI*, body mass index; *COPD*, chronic obstructive pulmonary disease; *NYHA*, New York Heart Association; *EuroSCORE*, European System for Cardiac Operative Risk Evaluation; *CK-MB*, creatine kinase-MB; *LVEF*, left ventricular ejection fraction.

∗ Median (Q1, Q3); n (%).

† Wilcoxon rank sum test; Pearson χ^2^ test; Fisher exact test.

**Table E2:** Intraoperative characteristics together with the overall cohort

Characteristic	Overall, N = 3867∗	BITA		*P* value†	Overall BITA N = 2010∗	Sex		*P* value†
		BITA, n = 2010∗	Non-BITA, n = 1857∗			Female, n = 268	Male, n = 1742∗	
No. of bypasses	3.00 (3.00, 4.00)	4.00 (3.00, 4.00)	3.00 (3.00, 4.00)	<.001	4.00 (3.00, 4.00)	3.00 (3.00, 4.00)	4.00 (3.00, 4.00)	.2
Complete revascularization	2675 (74%)	1441 (78%)	1234 (70%)	<.001	1441 (78%)	201 (80%)	1240 (77%)	.300
No. of affected vessels	3 (3, 3)	3 (3, 3)	3 (3, 3)	<.001	3 (3, 3)	3 (3, 3)	3 (3, 3)	.8
Time of cardiopulmonary bypass, min	90 (72, 107)	87 (70, 104)	92 (75, 112)	<.001	87 (70, 104)	85 (68, 99)	88 (71, 105)	.025
Time of aortic crossclamping, min	69 (54, 85)	69 (55, 85)	68 (53, 85)	.3	69 (55, 85)	68 (53, 80)	69 (55, 85)	.031
Total time of surgery, min	228 (195, 260)	240 (201, 262)	214 (180, 250)	<.001	240 (201, 262)	230 (200, 260)	240 (205, 262)	.12
LITA diameter, mm	2.80 (2.50, 3.00)	2.80 (2.50, 3.00)	2.80 (2.50, 3.00)	.6	2.80 (2.50, 3.00)	2.70 (2.35, 3.00)	2.90 (2.50, 3.00)	<.001
RITA diameter, mm	2.90 (2.50, 3.10)	2.90 (2.50, 3.10)	2.90 (2.50, 3.20)	.6	2.90 (2.50, 3.10)	2.70 (2.40, 3.00)	2.90 (2.50, 3.10)	<.001
Segment 1, %	50 (25, 99)	50 (25, 90)	63 (25, 100)	<.001	50 (25, 90)	50 (25, 99)	50 (25, 90)	.4
Segment 2, %	82 (38, 100)	80 (38, 100)	90 (38, 100)	<.001	80 (38, 100)	82 (38, 100)	75 (38, 100)	.8
Segment 3, %	50 (25, 90)	50 (25, 90)	50 (25, 90)	.2	50 (25, 90)	38 (13, 83)	50 (25, 90)	.3
Segment 4dp, %	25 (12, 75)	25 (12, 75)	25 (12, 75)	.11	25 (12, 75)	13 (0, 62)	25 (12, 75)	.017
Segment 4pL, %	25 (0, 63)	25 (0, 75)	25 (0, 62)	.3	25 (0, 75)	25 (12, 62)	25 (0, 75)	.6
Segment 5, %	38 (12, 75)	38 (12, 75)	38 (10, 75)	.7	38 (12, 75)	25 (0, 70)	38 (12, 75)	.056
Segment 6, %	70 (38, 83)	70 (38, 83)	70 (34, 90)	.6	70 (38, 83)	63 (25, 90)	75 (38, 83)	.4
Segment 7, %	75 (50, 90)	75 (50, 90)	75 (50, 90)	.2	75 (50, 90)	75 (50, 90)	75 (50, 90)	.8
Segment 8, %	25 (12, 62)	25 (12, 62)	25 (12, 52)	>.9	25 (12, 62)	25 (12, 62)	25 (12, 62)	.3
Segment 9a, %	63 (25, 83)	63 (25, 83)	62 (25, 83)	.2	63 (25, 83)	62 (15, 83)	63 (25, 83)	.13
Segment 9b, %	25 (0, 75)	25 (0, 75)	25 (0, 75)	.6	25 (0, 75)	25 (12, 62)	25 (0, 75)	.6
Segment 10, %	0 (0, 13)	0 (0, 13)	0 (0, 13)	.4	0 (0, 13)	0 (0, 13)	0 (0, 13)	.8
Segment 11, %	50 (13, 83)	50 (13, 83)	38 (12, 82)	.041	50 (13, 83)	50 (13, 83)	50 (13, 83)	.6
Segment 12, %	38 (12, 82)	47 (12, 83)	38 (12, 80)	.056	47 (12, 83)	25 (0, 82)	50 (12, 83)	.053
Segment 13, %	70 (25, 90)	75 (25, 90)	63 (25, 90)	.2	75 (25, 90)	63 (25, 90)	75 (25, 90)	.15
Segment 14a, %	50 (13, 83)	50 (13, 83)	50 (13, 90)	.4	50 (13, 83)	32 (13, 80)	50 (13, 83)	.065
Segment 14b, %	25 (12, 82)	25 (12, 82)	25 (12, 82)	.5	25 (12, 82)	25 (12, 83)	25 (12, 82)	.7
Min. flow in LITA graft, mL/min					60 (40, 90)	43 (30, 83)	60 (40, 90)	.007
Min. Flow in RITA graft, mL/min					60 (35, 88)	60 (35, 80)	60 (36, 90)	.2

*BITA*, Bilateral internal thoracic artery; *LITA*, left internal thoracic artery; *RITA*, right internal thoracic artery.

∗ Median (Q1, Q3); n (%).

† Wilcoxon rank sum test; Pearson χ^2^ test.

**Table E3:** Summary table of our study findings

Outcome/analysis type	Women vs men: direction of difference	Statistical significance	Main interpretation
Composite end point (death or revascularization)			
Unweighted KM (full follow-up)	Greater risk in women	*P* = .010 (log-rank)	Crude analysis shows greater cumulative events in women.
Overlap-weighted KM (full follow-up)	Slightly greater but attenuated in women	*P* = .051 (log-rank)	After weighting, sex difference is small and no longer conventionally significant.
Overlap-weighted KM, 30-d landmark	No clear difference	*P* = .139 (log-rank)	After excluding 30-d events, long-term risk appears similar by sex.
RSF permutation importance (composite end point)	Different leading predictors by sex	Not applicable (importance scores)	Distinct sex-specific risk profiles rather than a uniform sex effect.
All-cause mortality			
Overlap-weighted/landmark KM	Difference reduced or absent	(Insert)	After weighting and landmarking, sex differences in mortality are small or nonsignificant.
Fine-Gray regression (death)	Modest or no independent effect of sex	*P* value: .29/landmark *P* value: .120	Female sex not a strong independent predictor of death after adjustment.
RSF permutation importance (death)	Different drivers by sex	Not applicable	Similar pattern of sex-specific predictors as for composite end point.
Coronary revascularization			
Overlap-weighted/landmark KM	Attenuated differences	*P* value: .51/landmark *P* value: .65	After weighting/landmarking, revascularization rates are more similar between sexes.
Fine-Gray regression (revascularization)	No strong independent sex effect	*P* value: .075	Sex not a major independent predictor of repeat revascularization.
RSF permutation importance (revascularization)	Sex-specific predictors	Not applicable	Mechanisms for revascularization differ between sexes.

*KM*, Kaplan-Meier; *RSF*, random survival forest.

## Appendix E1

### Multiple imputation and weighing

Missing data affected several baseline and intraoperative variables, including graft diameters and intraoperative graft flow, with per-variable missingness ranging from 0% to 10%. Some degrees of missingness were greater, at approximately 20% (left internal thoracic artery/right internal thoracic artery [LITA/RITA] diameter). To handle missing data and ensure robust adjustment for confounders, multiple imputation was performed using the MICE (multiple imputation by chained equations) algorithm using the mice R Package (Version 3.17.0), incorporating clinically relevant baseline variables into the imputation model. We applied MICE to age, body mass index, diabetes, dyslipidemia, arterial and pulmonary hypertension, arrhythmia, smoking status, peripheral artery disease, chronic obstructive pulmonary disease, previous myocardial infarction, previous stroke, New York Heart Association class, dialysis, malignancy, number of bypasses, extent of coronary disease, and LITA/RITA diameters. Propensity scores for each patient were then estimated via logistic regression using the MatchIt R Package (version 4.7.1), modeling biological sex as a function of relevant clinical and procedural characteristics. Overlap weighting (11) was applied on the basis of these propensity scores, creating balanced comparison groups with respect to baseline characteristics, as confirmed by standardized mean differences visualized via Love plots (Figure E1).

### Multivariable regression analysis

Covariate selection for the multivariable Fine-Gray models followed a 2-step approach: variables associated with the composite end point at *P* < .10 in univariable analyses were considered, and the final models included clinically relevant covariates: age, hypertension, smoking, diabetes, previous stroke, arrhythmia, previous myocardial infarction, pulmonary hypertension, peripheral artery disease, chronic obstructive pulmonary disease, New York Heart Association class, and LITA and RITA graft flow.

### Random Survival Forest (RSF)

RSF modeling was used to analyze survival and revascularization outcomes among patients stratified by sex, offering a flexible, nonparametric approach to risk prediction in time-to-event data. Separate RSF models were constructed for male and female patients, with model training by 5-fold cross-validation and hyperparameter optimization conducted through randomized grid search for key parameters, including number of trees, minimal split size, minimal leaf size, and maximum tree depth. Model performance and discrimination abilities were evaluated using the concordance index (C-index).

The RSF models were fitted to each sex group after appropriate preprocessing and splitting of the dataset into training and test sets. For each sex, the target variable was formulated as time-to-composite end point. Posttraining, permutation importance scores were calculated by randomly permuting each predictor in the test set and quantifying the associated decrease in model concordance; larger decreases indicate greater variable importance for prediction. This allowed ranking of variable importance specifically for male and female patients.

To evaluate model calibration, predicted survival probabilities at 5 years were grouped into deciles across the test data. Within each probability bin, observed event probabilities were calculated using the Kaplan-Meier estimator, generating empirically based calibration curves. Calibration was visualized for both sexes by plotting predicted probability against observed probability (Figure E2, Figure E3, Figure E4). All plots were created in Python using matplotlib. The final c-score of the model was 0.65 for composite end point, 0.67 for death and 0.64 for revascularization. Calibration plots indicated deviations from ideal calibration, particularly in extreme risk deciles, consistent with the limited number of events within sex-stratified models and the relative complexity of the RSF; accordingly, these models were interpreted as exploratory.
